# Rapid detection of haemotropic mycoplasma infection of feline erythrocytes using a novel flow cytometric approach

**DOI:** 10.1186/1756-3305-6-158

**Published:** 2013-05-31

**Authors:** Angeles Sánchez-Pérez, Graeme Brown, Richard Malik, Stephen J Assinder, Katherine Cantlon, Christine Gotsis, Samantha Dunbar, Stuart T Fraser

**Affiliations:** 1 Discipline of Physiology and Bosch Institute, School of Medical Sciences, University of Sydney, Medical Foundation Building K25, 92-94 Parramatta Road, Camperdown, NSW, 2050, Australia; 2 Faculty of Veterinary Science, B14, University of Sydney, Camperdown, NSW, 2006, Australia; 3 Centre for Veterinary Education Conference Centre, B22, University of Sydney, Camperdown, NSW, 2006, Australia; 4 Molecular Department, Gribbles Veterinary Pathology, Glenside, SA, 5065, Australia; 5 Discipline of Anatomy and Histology, School of Medical Sciences, University of Sydney, Camperdown, NSW, 2006, Australia

**Keywords:** Hemoplasma, Hemotropic mycoplasma, Flow cytometry, Diagnosis

## Abstract

**Background:**

The haemotropic mycoplasmas *Mycoplasma haemofelis* and *Candidatus* Mycoplasma haemominutum cause feline infectious anaemia with infection rates in feline populations reflecting widespread subclinical infection. Clinically significant infections are much rarer but can be life-threatening. Current diagnosis is dependent upon visualising organisms in stained blood smears, PCR or quantitative PCR (qPCR). These procedures are labour-intensive and time-consuming. Furthermore, PCR-based approaches offer limited insight into the disease burden of the infected animal.

**Methods:**

We have developed a novel and rapid flow cytometric system that permits diagnosis of haemotropic mycoplasma infections and quantitation of the percentage of erythrocytes that are parasitized. The method exploits the fact that mature mammalian erythrocytes, the host cell for haemoplasmas, are enucleated and thus lack nucleic acid. DRAQ5 is a synthetic anthrocycline dye which rapidly crosses cell membranes and binds to nucleic acids. The presence of exogenous bacterial DNA in mammalian erythrocytes can, therefore, be detected by DRAQ5 uptake and flow cytometric detection of DRAQ5 fluorescence.

**Results:**

Here, we show that this system can detect epi-erythrocytic infection of companion felines by haemotropic mycoplasma. Due to their differences in size, and hence the quantity of DNA, the two major feline hemoplasmas *M. haemofelis* and *Candidatus* M. haemominutum can be distinguished according to DRAQ5 fluorescence. We have also shown the usefulness of DRAQ5 uptake in monitoring a cat infected with *M. haemofelis* sequentially during treatment with doxycycline.

**Conclusions:**

The technique described is the first report of a flow cytometric method for detecting haemotropic mycoplasmas in any species and could be applied to widespread screening of animal populations to assess infection by these epi-erythrocytic parasites.

## Background

Mammalian erythroid cells mature in a progressive manner from erythroblasts (possessing a nucleus), to anuclear reticulocytes bearing RNA, and finally to mature erythrocytes lacking both a nucleus and RNA transcripts. This makes them susceptible to a range of parasitic organisms, such as malarial *Plasmodium* and *Babesia sp.* in both humans and animals. Haemoplasmas such as the haemotropic *Mycoplasma haemofelis* (Hf), *Candidatus* Mycoplasma haemominutum (Hm) and *Candidatus* Mycoplasma turiensis can infect erythrocytes and are the most common cause of feline infectious anaemia (FIA) worldwide [1]. Haemotropic mycoplasmas (also called haemoplasmas), previously classified as *Haemobartonella* species, attach themselves to the surface of feline erythrocytes, inducing changes in the shape and surface texture of the host erythrocyte thereby enhancing their clearance by mononuclear phagocytes in the spleen [2,3]. Coomb’s-positive cells are often seen in cats with high levels of infection, suggesting that antibody-induced extravascular haemolysis may also occur [4]. While healthy cats can generally tolerate a low burden of epi-erythrocytic mycoplasmas, cats that are immunologically naive or immunocompromised may develop anaemia [5], which in some instances become life-threatening.

The current “gold standard” diagnostic test is based on PCR analysis of mycoplasma 16S ribosomal RNA gene in peripheral blood samples [6,7], although in some patients’ pathogens can be visualised in routine blood films stained with Romanovsky-type stains such as Giemsa, Wright, or DiffQuik stains. While this is useful in determining the species of mycoplasma infecting the patient, it does not quantify the proportion of erythrocytes that are parasitised. With this in mind, we have developed a novel flow cytometric system that permits analysis of large numbers of erythrocytes very rapidly, while assessing whether nucleic acid (which can only be derived from adherent mycoplasma) is present or absent in or on the cat’s red blood cells. We have capitalised on the rapid cell-permeability of the synthetic anthrocycline DNA-binding dye DRAQ5 to demonstrate the presence or absence of erythrocytes bearing Mycoplasma, as evidenced by the presence of nucleic acid, which can only originate from exogenous microorganisms. DRAQ5 uptake has been previously used to segregate nucleated and anuclear erythroid cells in the developing mouse embryo and distinguish haematopoietic cell types in the bone marrow [8,9]. This system has allowed us to analyse numerous clinical samples in a fraction of the time required for blood smear analysis, and also to quantify the level of parasitaemia of red blood cells (RBCs) in a representative number of subclinical naturally-occurring infections, as well as during treatment of acute life-threatening disease.

Haemotropic mycoplasma infection has recently been detected in human patients with anaemia [10,11]. Given the abundance of feline and canine pets and their close living relationship with humans, the danger of a putative human mycoplasma zoonosis requires the development of quick and effective diagnostic methods. This was exemplified by the recent diagnoses of human patients with co-infections of haemotropic *Mycoplasma sp* as well as *Bartonella sp*[12]. One of these patients presented with simultaneous co-infection with *Anaplasma platys*, a rickettsial pathogen that resides in mammalian platelets [13]. Here, we present our findings that may offer a novel system for identifying pathogens residing within mammalian erythrocytes, and in the future, could be adapted to pathogens targeting mammalian platelets.

## Methods

### Clinical specimens

Cat blood samples were obtained from Veterinary Pathology Diagnostic Services, Faculty of Veterinary Science, The University of Sydney; Paddington Cat Clinic, Double Bay Veterinary Clinic; and Gribbles Veterinary Pathology, the sole laboratory in Australia offering qPCR diagnosis for feline haemotropic mycoplasmas at the time of these investigations. Blood was collected from the cephalic or jugular veins (using gentle physical restraint, sedation or while under general anaesthesia) as part of routine clinical specimen collection.

Samples from the Gribbles Laboratory belong to four groups. Samples in Group 1 had been identified by PCR as being negative for mycoplasma (5 cats). These cats were anaemic and qPCR had been requested to identify if feline infectious anaemia was present. Groups 2 and 3 were PCR-positive for *Candidatus* M. haemominutum (27 cats) or *M. haemofelis* (7 cats), respectively. Group 4 corresponded to cats showing no signs of anaemia but had peripheral blood samples collected in the diagnosis of other health issues. PCR identification of haemotropic *Mycoplasma sp* was performed as reported previously [7].

### Flow cytometry

Presence of exogenous mycoplasma DNA on the RBCs was analysed by flow cytometry using the DRAQ5 nuclear dye (Alexis Biochemicals, Lausanne, Switzerland). Samples of peripheral feline blood (10 μl) were incubated with 5 μM DRAQ5 in PBS for 1 min at room temperature (RT), washed and suspended in 200 μl of PBS. No cell lysis or permeabilisation is required for DRAQ5 uptake. Flow cytometric analyses were performed, and at least 500,000 cells counted, using a FACSCalibur (Becton Dickinson, San Jose, CA, USA) and CellQuest software (Becton Dickinson). The FACSCalibur used in this study allows a flow rate of approximately 9,000 events per second, thus allowing us to assess 500,000 cells within one minute. Half a million cells were routinely analysed for the studies presented here. To optimise this fluorescent labelling technique, 10 μl of venous blood was incubated with 5 μM DRAQ5 for 1, 2, 5 or 10 min at room temperature and then analysed. One minute was sufficient for DRAQ5 uptake and binding to any nucleic acid associated with the erythrocytes (data not shown). Therefore, 1 min was chosen as the optimal staining period. Data was analysed using the FlowJo software package (Treestar, San Carlos, CA, USA) [8]. Significant differences in the uptake of DRAQ5 between group means were tested by one-way analysis of variance (ANOVA).

## Results

### Detection of nucleic acid-bearing feline erythrocytes by flow cytometry

The first question we addressed was whether feline haemotropic mycoplasmas can be detected by flow cytometry via fluorescent labelling of nucleic acid. Peripheral blood samples from cats that had been screened by qPCR for the presence or absence of *Candidatus* M. haemominutum were incubated with DRAQ5 for 1 minute and analysed immediately by using flow cytometry. “Control” specimens form normal cats presented for routine neutering at veterinary clinics were also examined using the same methodology.

**Figure 1:**
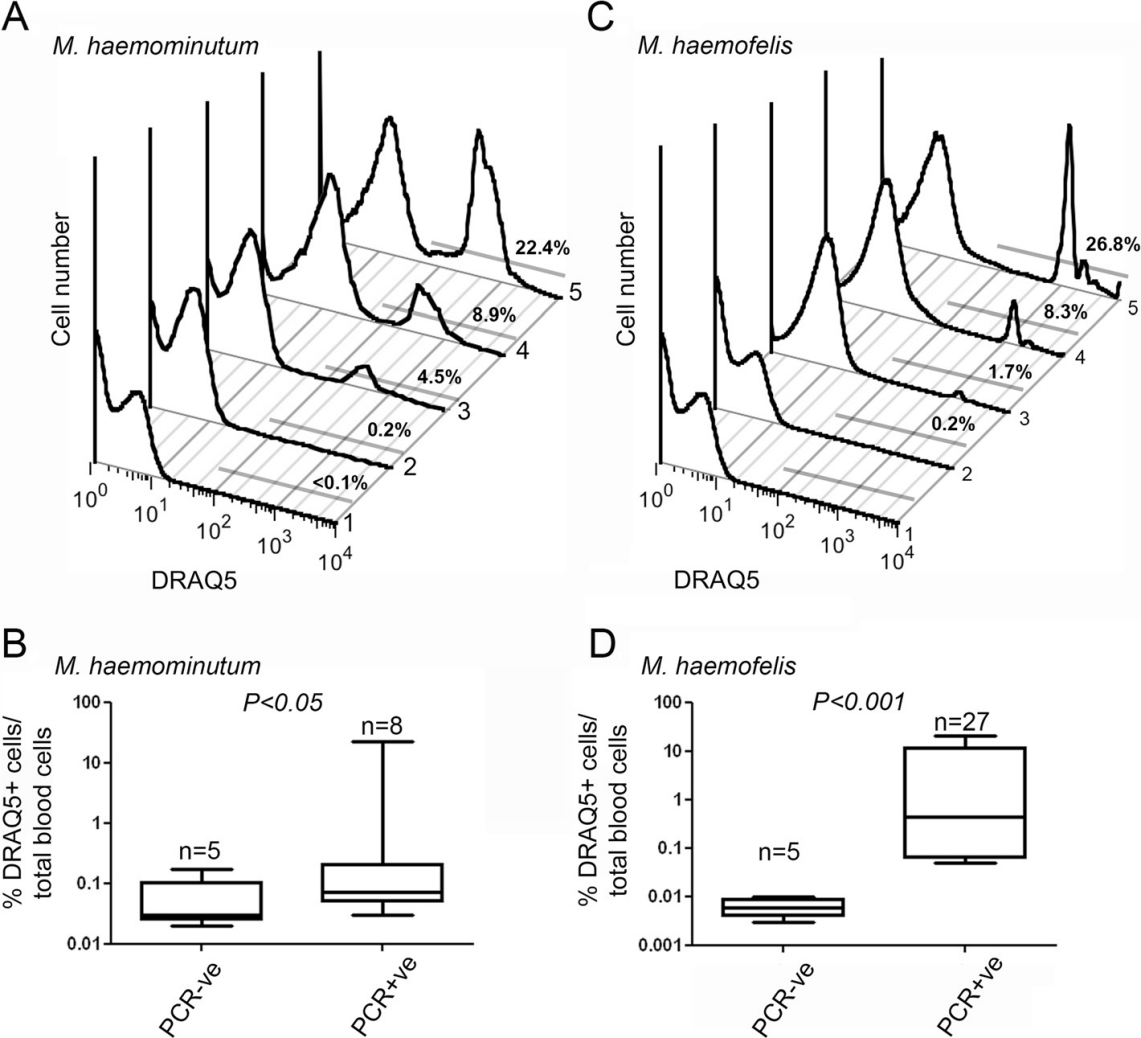
**Feline haemoplasma infection can be detected by flow cytometric analysis of peripheral blood cells obtained from cats.** Representative flow cytometric profiles showing DRAQ5 uptake in five peripheral blood samples (1–5). Samples from domestic cats were first assessed by PCR for infection with *Candidatus* Mycoplasma haemominutum (**A**) or *Mycoplasma haemofelis* (**C**). Histogram 1 shows a lack of DRAQ5-positive cells in the blood of an uninfected (PCR-negative) cat, whereas histograms 2–5 show DRAQ5-uptake in blood cells in a number of infected (PCR-positive) cats. The grey bar indicates the region containing DRAQ5-positive cells. The numbers above the bar indicate the frequency of DRAQ5-positive cells out of total live gated peripheral blood cells. Box-and-whisker plots showing the range of DRAQ5-uptake of the amount of DRAQ5 present in blood samples from uninfected (PCR-negative) cats (n=5) or patients infected with either *Candidatus* M. haemominutum (**B**, n=8) or M. haemofelis (**D**, n=27), expressed as a logarithmic plot of the percentage of DRAQ5-positive cells. P values demonstrate significant differences between group means as by one-way ANOVA.

As can be seen in Figure 1A and C, PCR-negative samples showed very low levels of DRAQ5-positive cells. In contrast, samples determined to be PCR-positive for *Candidatus* M. haemominutum showed that a broad range of cells had taken up DRAQ5 (Figure 1A). While some PCR-positive samples showed levels of DRAQ5 only slightly higher than the uninfected control, other PCR-positive samples showed DRAQ5 uptake in over 20% of circulating blood cells, with fluorescence intensity clearly above the background levels seen in healthy animals. A statistically significant difference in DRAQ5 uptake was observed between animals that were PCR-negative and PCR-positive for *Candidatus* M. haemominutum (Figure 1B). Indeed, screening of outwardly normal cats confirmed that healthy cats have a very low percentage (<0.01%) of DRAQ5(+) cells.

Having successfully detected increased DRAQ5 uptake in erythrocytes from cats infected with *Candidatus* M. haemominutum we next asked whether we could use this system to detect infection by another feline haemoplasma, *M. haemofelis*. Similar to the previous results, a range of DRAQ5-uptake was observed in *M. haemofelis* PCR-positive cats. Indeed, we observed a succession of DRAQ5+ve peaks suggesting that some cells were infected with higher levels of *M. haemofelis*, resulting in greater uptake of the DNA-binding dye. Analysis of 27 different cats infected with *M. haemofelis* showed a clearly significantly higher level of DRAQ5 uptake compared to PCR-negative cats (Figure 1D).

**Figure 2:**
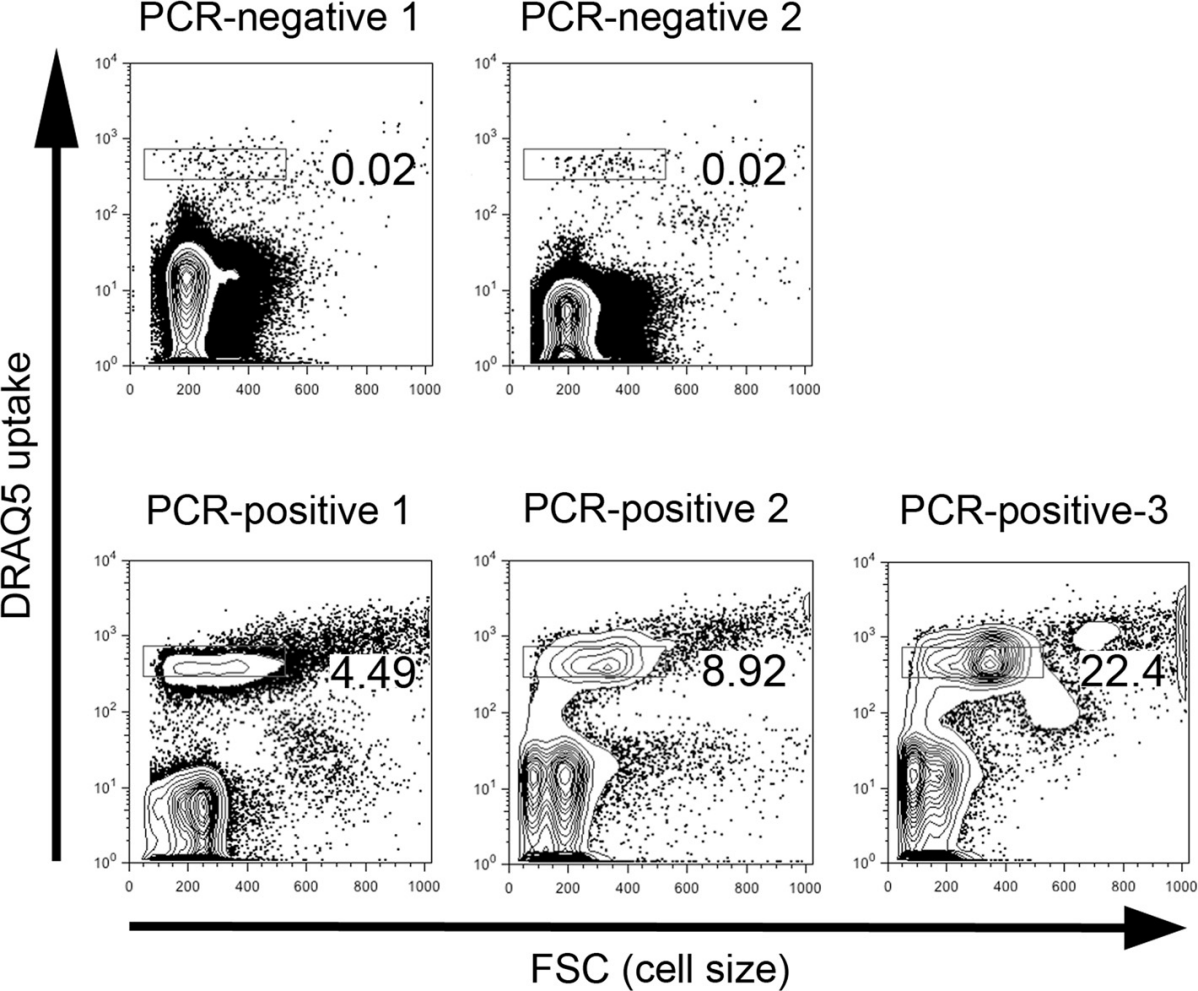
**Epierythrocytic infection of feline peripheral blood as detected by DRAQ5 uptake.** Flow cytometric analyses of representative peripheral blood samples from cats determined by PCR to be uninfected (PCR-negative) or infected (PCR-positive) with *Candidatus* Mycoplasma haemominutum. (A) Flow cytometric profiles show DRAQ5 uptake (Y-axis) compared to cell size (FSC: X-axis). The rectangular gates correspond to the presence of infected erythrocytes, with the number corresponding to the frequency of infected cells out of total live, gated cells. The majority of cells with high DRAQ5 uptake are small, indicative of erythrocytes.

The next question we addressed was whether the cells that took up high levels of DRAQ5 were erythrocytes. Flow cytometry permits for analysis of the size and granularity of cells. Erythrocytes are smaller than all other haematopoietic cells and lack cytoplasmic granules. As seen in Figure 2, the cells that showed high levels of DRAQ5 signal were predominantly small cells, fitting the reported size range for feline erythrocytes. However, there was a subset of cells that were larger than erythrocytes, possibly representing reticulocytes or other blood cells such as granulocytes or lymphocytes.

### *M. haemofelis* and *Candidatus* M. haemominutum infection of feline erythrocytes can be distinguished by DRAQ5 fluorescence intensity

**Figure 3:**
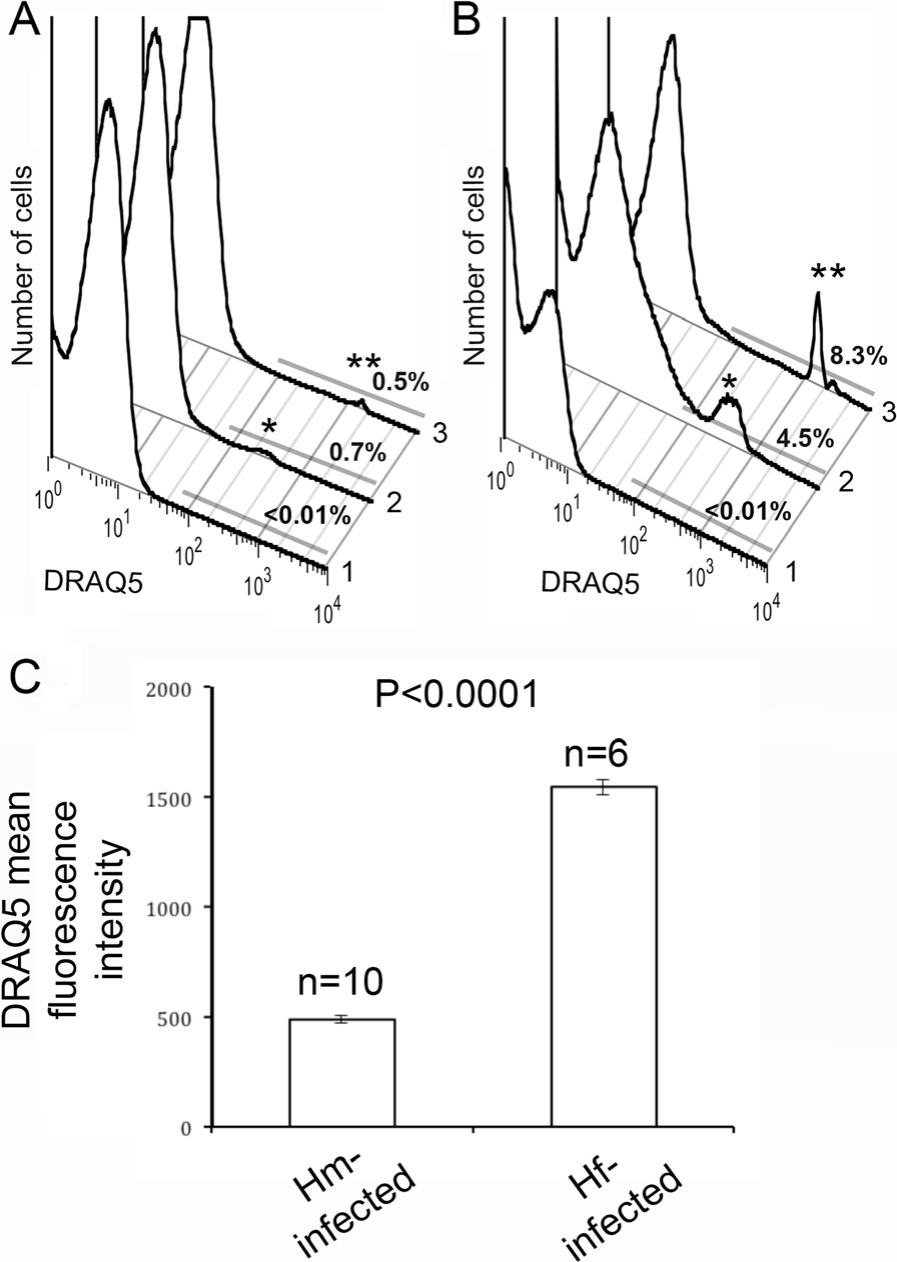
**Feline haemoplasma can be identified according to DRAQ5-uptake and flow cytometric detection.** The two major feline haemoplasmas *Candidatus* Mycoplasma haemominutum and *Mycoplasma haemofelis* can be identified according to the fluorescence intensity of DRAQ5-uptake by infected erythrocytes. The foremost histogram is representative of DRAQ5-uptake in an uninfected animal. In a screening examination of clinically healthy cats, low levels of DRAQ5-uptake are detected showing the presence of few infected cells (**A**, **B**, histogram 1). Mean fluorescence intensity is lower in the cat infected with the smaller haemoplasma (M. haemominutum, histogram 2, peak indicated by *) compared to the cat infected with the larger M. haemofelis (histogram 3, peaks indicated by **). The difference in DRAQ5-uptake is more clearly seen in animals with higher level of parasitaemia (**B**) (*Candidatus* M. haemominutum, histogram 2, peak indicated by *; M. haemofelis (histogram 3, peaks indicated by **). (**C**) The mean fluorescent intensity from *Candidatus* M. haemominutum-infected erythrocytes (n=10) is clearly lower than that of erythrocytes from animals that have been tested by PCR to be infected by M. haemofelis (n=6). ANOVA was performed to demonstrate the significant difference in mean fluorescence intensities between animals infected with *Candidatus* M. haemominutum or M. haemofelis.

*M. haemofelis* is approximately 2-fold larger than *Candidatus* M. haemominutum [14] and should contain more DNA and take up greater amounts of DRAQ5. As PCR genotyping of mycoplasma species infecting the different feline samples had been performed, we could compare the levels of DRAQ5 uptake by erythrocytes infected with these different haemoplasmas. We repeatedly observed higher levels of DRAQ5 uptake in *M. haemofelis*-infected samples compared to *Candidatus* M. haemominutum-infected cells, as observed with samples showing low levels of infection assessed in the same experimental study (Figure 3A); and also observed in samples with higher levels of infection assessed at different time points (Figure 3B). The mean fluorescent intensity of DRAQ5-positive cells from 10 different *Candidatus* M. haemominutum PCR-positive samples was 487 (Figure 3C) fluorescent units, whereas the main population of DRAQ5-positive cells from 6 different *M. haemofelis* PCR-positive samples was 1544, a 3-fold brighter DRAQ5 signal (Figure 3C). These findings support a preliminary diagnosis of the causative agent for FIA using flow cytometry.

### Flow cytometric monitoring of haemoplasma infection

**Figure 4:**
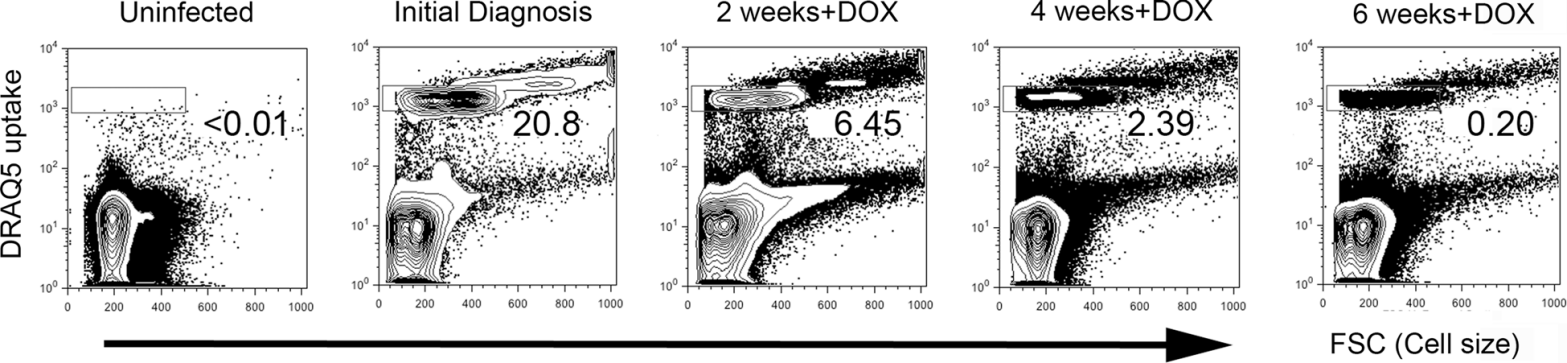
**Flow cytometric monitoring of haemoplasma infection during treatment using doxycycline.** Blood samples from an anaemic cat with a *Mycoplasma haemofelis* (Hf) infection with were taken at the point of initial diagnosis and every two weeks following commencement of doxycycline therapy. Flow cytometric profiles show DRAQ5 uptake versus cell size (FSC). The rectangular gates correspond to the location of *Mycoplasma haemofelis*-infected erythrocytes, with numbers indicating the frequency of DRAQ5-positive erythrocytes. Left panel: DRAQ5-uptake in an uninfected animal. Subsequent panels show DRAQ5-uptake in samples from the same animal from initial diagnosis and at 2-weekly intervals following commencement of doxycycline (DOX) treatment. The frequency of DRAQ5-positive erythrocytes clearly decreases over time with successful antimicrobial therapy.

We tested our flow cytometric system further with samples collected from a neutered male cat that presented with regenerative anaemia with mild reticulocytosis. A *M. haemofelis* infection was diagnosed using qPCR. This animal was treated with doxycycline monohydrate (5 mg/kg twice daily), the standard recommended treatment for feline infectious anaemia attributable to haemotropic mycoplasmas. Blood samples were collected at the first clinical visit and subsequently every two weeks after commencing treatment, and analysed for DRAQ5 uptake by erythrocytes. The patient had a high percentage of infected erythrocytes at the time of admission, with almost 21% of erythrocytes taking up DRAQ5 (Figure 4). Interestingly, a substantial number of larger cells were present in the circulation that had very high levels of DRAQ5-uptake. This level of uptake is higher than typically seen for DRAQ5 binding to RNA in reticulocytes, suggesting that reticulocytes were infected by *M. haemofelis* in this cat. The proportion of infected erythrocytes decreased to less than half within two weeks of commencing doxycycline, with a more dramatic decrease occurring over the next four weeks of treatment. At six weeks post-treatment, the cat had numbers of DRAQ5-positive erythrocytes 700-fold less than at the time of admission.

## Discussion

A range of laboratory techniques have been proposed for the diagnosis of feline haemoplasma infection in cats with anaemia including blood smear analysis, serological testing by Western blot [15] and qPCR [6]. We describe here a fast, accurate and reliable method for determining the parasitic burden of haemotropic mycoplasmas in cats. This method relies on the use of DRAQ5, a synthetic anthrocycline dye that rapidly (within 1 min) crosses cell membranes and binds to nucleic acids. DNA is bound with higher affinity than RNA and generates a more intense fluorescence signal upon excitation in a flow cytometer. As shown here, our method reliably identifies the two principal feline parasitic mycoplasma species (*M. haemofelis* and *Candidatus* M. haemominutum) and potentially distinguishes between them. Our approach allows for rapid detection of patients with a high parasite burden, thus identifying those anaemic cats requiring immediate antimicrobial therapy. It has the additional advantage of only requiring a few microlitres of EDTA-treated blood. In contrast, the current PCR identification method is much more labour-intensive, requiring DNA extraction and purification followed by DNA amplification and gel analysis of the PCR product. While this technique does require the presence of a flow cytometer with a red laser capable of exciting DRAQ5, and appropriate technical expertise, such equipment is becoming more readily available in clinics, diagnostic units or universities. The use of a single fluorescent dye to detect mycoplasmas adhered to erythrocytes makes this a simple flow cytometric protocol.

At least two distinct haemoplasmas can act as the causative agent of feline infectious anaemia. Due to the size differences between the haemoplasmas, DRAQ5 labelling is stronger for *M. haemofelis*-infected erythrocytes than for those harbouring *Candidatus* M. haemominutum. This, in turn, results in a much higher fluorescence signal and hence a lower DRAQ5 fluorescent background in the former, which makes low levels of *M. haemofelis* much easier to identify than low levels of *Candidatus* M. haemominutum. Some of the samples analysed had been refrigerated at 5°C for up to two months, yet still displayed a strong DRAQ5 uptake signal. This was surprising, as it has been previously suggested that haemotropic mycoplasmas detach from the surface of feline erythrocytes during incubation in the presence of heparin or EDTA [15]. However, we reproducibly observed a strong DRAQ5 signal from cats that had earlier been confirmed by PCR to be infected. Parasitaemia of erythrocytes by mycoplasmas can be highly variable with rapid changes in the level of bacterial adherence over a period as short as a few hours. Possibly, samples may have originally had even higher levels. Alternatively, a subset of parasites may be more strongly attached than others. As we routinely assess infection in 500,000 cells, it is possible that our greater coverage of cells is more likely to detect mycoplasmas that remain attached. Whilst a number of haemotropic mycoplasmas can infect feline erythrocytes, cats are more commonly infected with a single pathogenic species [16]. Hence, fluorescent signal obtained by DRAQ5 uptake is likely to represent a single pathogen in most instances.

Haemoplasmas can also infect erythrocytes in other mammalian species including dogs [17,18]. *Mycoplasma suis* and *M. parvus* are the causative agents of swine hemoplasmosis, a condition leading to deaths in young animals, chronic infection, failure to thrive and significant economic costs [19]. Likewise, *M. wenyonii* and *M. haemobos* have been implicated as a cause of anaemia in cattle and M. ovis is a common cause of acute mortality in sheep. As DRAQ5 uptake is passive and not species-specific, this system could be used in diagnosing and monitoring haemoplasmosis in these important production animals. Recent reports have demonstrated that haemoplasma can also infect human erythrocytes. While initially this observation was made in an immunocompromised patient [10], similar observations have been made in an individual with no discernible immunodeficiency [11,12]. This simple flow cytometric system, possibly in combination the numerous fluorescently-conjugated antibodies available against human erythrocytes, could serve as a diagnostic tool for patients thought to present with haemoplasmosis. The system described here could, in the future, therefore assist in the diagnosis of zoonotic haemoplasmosis. The speed and economy of the technique would lend itself to the routine screening of human populations at risk of zoonotic transmission due to lifestyle and exposure to risk factors, for example agricultural workers at risk of developing *M. ovis* through contamination of minor abrasions by sheep blood during shearing or lamb marking.

We have recently applied this system to assessing other parasites known to infect mammalian erythrocytes. DRAQ5 was extremely useful in assessing infection of mouse erythrocytes by *Plasmodium falciparum* in an *in vivo* model. Likewise, distinct stages of malarial parasite burden could be identified using DRAQ5 and flow cytometry in cultures of human erythrocytes (Sanchez-Perez et al., manuscript in submission). In preliminary studies DRAQ5 uptake has also recently been used to examine infection of canine erythrocytes with the apicomplexan parasite *Babesia canis*. We aim to explore the utility of this system further to determine whether other erythrocytic parasites such as *Hepatozoon* species [20] can be readily observed. DRAQ5 fluorescence is being assessed as a possible indicator of intra-platelet infection by *Anaplasma platys* in tick-infested dog blood samples.

The use of PCR and subsequently qPCR revolutionised the diagnosis of feline infectious anaemia, and provided a vital research tool to unravel the kinetics of haemoplasma infection in mammalian hosts. We believe the method described herein will provide an additional layer of information which will inform on the kinetics of infection, by determining the extent of parasitaemia (i.e. the proportion of infected cells) as well as the number of organisms per unit volume of blood. The technique requires so little blood that studies of needle aspirates from the spleen and bone marrow might provide important new information on the fine detail of how this organism persists and multiplies within the mammalian host. Furthermore, its ability to detect all bacterial and likely protozoan haemoparasites lends itself to screening of large populations for occult or subclinical disease, without the requirement of knowing exactly which species will be involved in a given population.

## Conclusions

Feline infectious anaemia is a significant health issue for companion cats as well as captive nondomestic feline species. Diagnosis has relied upon cytological examination and confirmation by PCR. This is the first report utilizing flow cytometry to identify the type of haemoplasma infecting mammalian erythrocytes and allows the identification of the two major mycoplasmal species leading to FIA. This system also allows rapid enumeration of the disease burden facing the infected animal. Haemotropic mycoplasma infect a range of other mammalian species including humans. This system is not reliant upon the host species and hence could be easily adapted for the flow cytometric detection of haemoplasma in humans, livestock and other important mammalian species.

## Abbreviations

FIA: Feline infectious anaemia; Hm: *Candidatus* mycoplasma haemominutum; Hf: *Mycoplasma haemofelis*; PBS: Phosphate-buffered saline; PCR: Polymerase chain reaction; RBC: Red blood cell; RT: Room temperature

## Competing interests

The authors declare no competing interests.

## Authors’ contributions

ASP, GB, RM and STF conceived of the study. ASP performed flow cytometric and data analyses, prepared figures and drafted the manuscript. GM, RM, KC, CG and SD collected samples and carried out diagnostic tests. SJA performed statistical analyses on flow cytometric data. STF prepared figures and drafted the manuscript. All authors have read and approved the final version of this manuscript.
